# Correlated Dynamics in Ionic Liquids by Means of NMR Relaxometry: Butyltriethylammonium bis(Trifluoromethanesulfonyl)imide as an Example

**DOI:** 10.3390/ijms22179117

**Published:** 2021-08-24

**Authors:** Danuta Kruk, Elzbieta Masiewicz, Sylwia Lotarska, Roksana Markiewicz, Stefan Jurga

**Affiliations:** 1 Department of Physics and Biophysics, University of Warmia and Mazury in Olsztyn, Oczapowskiego 4, 10-719 Olsztyn, Poland; elzbieta.masiewicz@uwm.edu.pl (E.M.); sylwia.lotarska@uwm.edu.pl (S.L.); 2 NanoBioMedical Centre, Adam Mickiewicz University, Wszechnicy Piastowskiej 3, 61-614 Poznan, Poland; rokmar@amu.edu.pl (R.M.); stjurga@amu.edu.pl (S.J.)

**Keywords:** ionic liquids, relaxation, dynamics, diffusion, nuclear magnetic resonance

## Abstract

^1^H and ^19^F spin-lattice relaxation experiments have been performed for butyltriethylammonium bis(trifluoromethanesulfonyl)imide in the temperature range from 258 to 298 K and the frequency range from 10 kHz to 10 MHz. The results have thoroughly been analysed in terms of a relaxation model taking into account relaxation pathways associated with ^1^H–^1^H, ^19^F–^19^F and ^1^H–^19^F dipole–dipole interactions, rendering relative translational diffusion coefficients for the pairs of ions: cation–cation, anion–anion and cation–anion, as well as the rotational correlation time of the cation. The relevance of the ^1^H–^19^F relaxation contribution to the ^1^H and ^19^F relaxation has been demonstrated. A comparison of the diffusion coefficients has revealed correlation effects in the relative cation–anion translational movement. It has also turned out that the translational movement of the anions is faster than of cations, especially at high temperatures. Moreover, the relative cation–cation diffusion coefficients have been compared with self-diffusion coefficients obtained by means of NMR (Nuclear Magnetic Resonance) gradient diffusometry. The comparison indicates correlation effects in the relative cation–cation translational dynamics—the effects become more pronounced with decreasing temperature.

## 1. Introduction

Properties of condensed matter systems are determined by their structure and dynamics. In the case of ionic liquids, their conductivity properties result from the timescale and mechanism of ionic diffusion. To gain information about ionic diffusion, it is essential to determine the values of translation diffusion coefficients of the ions [1,2]. This is, however, only the first step, and it is far from being sufficient. There is a long list of questions that need to be answered in order to obtain a deep insight into the mechanism of ionic motion. 

The first one concerns the range of the translation diffusion in connection to the timescale of the motion. Even if local (short range) translation diffusion is fast, long range translation displacements might require longer times due to bottlenecks in the diffusion paths. Consequently, one should consider short- and long-range translation diffusion coefficients, being aware that conductivity depends on the long-range translation movement. In this context, it is worth noting that the determination of diffusion coefficients for slow dynamics poses a challenge due to the limitations of Nuclear Magnetic Resonance (NMR) gradient methods [3,4,5], referred to as NMR diffusometry. NMR diffusometry is a well-established method of measuring translation diffusion coefficients—it exploits a magnetic field gradient that allows to identify the position of molecules (ions) carrying NMR active nuclei (^1^H and ^19^F typically for ionic liquids) versus time, as a result of changes in the resonance frequency. The most important characteristic of NMR diffusometry is that this method provides values of self-diffusion coefficients [3,4,5], in contrast to NMR relaxometry, exploited in this work, which probes relative translation motion of ions (molecules).

The second question concerns diffusion paths, especially the dimensionality of the diffusion process. For ionic liquids in bulk, the translation diffusion is isotropic (three-dimensional) [6,7,8,9,10,11], but for ionic liquids in confinement (e.g., ionogels), one can expect geometrical restrictions that reduce the dimensionality of the motion [12,13]. 

Eventually, the third subject to be addressed is a correlation of the ionic motion. Correlated ionic displacements are an important factor influencing conductivity of electrolytes and, consequently, raising considerable interest [14].

Classical NMR experiments are performed at a single magnetic field (resonance frequency). At high magnetic fields, one can obtain a deep insight into fast dynamics—i.e., rotational and internal motion of molecular and ionic systems as a consequence of the general rule that at a given resonance frequency, the dominating contribution to the relaxation is associated with a dynamical process occurring on a timescale being of the order of the inverse resonance frequency. Although this statement should be treated with caution as one should account for the strength (amplitude) of spin interactions, in fact, at high resonance frequencies, one probes fast dynamics. Thanks to the Fast Field Cycling (FFC) technology in relaxometry experiments, one can vary the magnetic field in a broad range (typically from about 5 kHz to (10–40) MHz, referring to the ^1^H resonance frequency) [15,16]. Consequently, one can probe in a single experiment molecular (ionic) motion on the time scale from ms to ns. NMR relaxometry not only gives access to the value of the diffusion coefficients but also allows identification of the mechanism (dimensionality) of the motion [12,13,17,18,19,20,21]. 

According to spin relaxation theory, ^1^H (^19^F) relaxation rates are given as linear combinations of spectral density functions being Fourier transforms of corresponding correlation functions characterising the dynamical processes that give rise to stochastic fluctuations of magnetic dipole–dipole interactions causing the relaxation processes [22,23,24,25,26,27]. As the mathematical form of the correlation function (and, hence, the spectral density) depends on the mechanism of the motion [13,17,18,28,29,30,31], the relaxation dispersion profiles (spin-lattice relaxation rates versus the resonance frequency) are a direct fingerprint of this mechanism. In this way, one can unambiguously distinguish between translational and rotational dynamics and reveal the isotropy/anisotropy of the motion [32,33]. 

As already anticipated, relaxation processes are caused by mutual, inter-molecular (inter-ionic) magnetic dipole–dipole interactions and, consequently, NMR relaxometry gives access to a relative translation diffusion of these species. In the case of uncorrelated motion, the relative translation diffusion coefficient is given as a sum of self-diffusion coefficients of the interacting species; for identical molecules (ions), the relative diffusion coefficient is twice as large as the self-diffusion one. This relationship breaks down in the case of a correlated motion. Consequently, NMR relaxometry offers the unique advantage of revealing correlated ionic dynamics.

NMR relaxometry has been applied to investigate dynamical properties of both, molecular and ionic liquids. To our knowledge, the subject of a correlated translation movement of ions probed by means of NMR relaxometry has, however, not been discussed. The major difficulty when addressing this subject lies in the challenging theoretical modelling of relaxation processes in systems containing various NMR active nuclei (such as ^1^H and ^19^F). To enquire into the dynamical properties of ^1^H containing cations and ^19^F containing anions in ionic liquids, one has to properly model ^1^H and ^19^F relaxation processes accounting for the role of ^1^H–^19^F (cation–anion) magnetic dipole–dipole interactions.

In this work, we present a thorough analysis of ^1^H and ^19^F spin-lattice relaxation data butyltriethylammonium bis(trifluoromethanesulfonyl)imide ([TEA-C4][TFSI]), taking into account all relevant relaxation pathways, especially the role of ^1^H–^19^F (cation–anion) mutual dipole–dipole coupling. In this way, we quantitatively describe the translational and rotational dynamics of the ions and enquire into correlation effects in the translation movement. Consequently, the work has two intertwined goals: to present the methodology that enables probing translation diffusion of ions in ionic liquids by means of NMR relaxometry and to reveal the scenario of the translation movement in [TEA-C4][TFSI]. A deep insight into the dynamical properties of ionic liquids is necessary for revealing factors determining conductivity of liquid electrolytes and, consequently, their tailoring for specific applications.

## 2. Theory

^1^H and ^19^F relaxation processes are caused by magnetic dipole–dipole interactions that can be of intra-molecular (intra-ionic) and inter-molecular (inter-ionic) interactions. For ionic liquids composed of ^1^H containing cations and ^19^F containing anions, the ^1^H and ^19^F spin-lattice relaxation rates, $R_{1,H}\left(\omega_{H}\right)$ and $R_{1,F}\left(\omega_{F}\right)$, respectively, ($\omega_{H}$ and $\omega_{F}$ denote ^1^H and ^19^F resonance frequencies, respectively, in angular frequency units), include the following relaxation contributions:

(1) $$R_{1,H}\left(\omega_{H}\right)=R_{1,H}^{intra}\left(\omega_{H}\right)+R_{1,H}^{inter,HH}\left(\omega_{H}\right)+R_{1,H}^{inter,HF}\left(\omega_{H}\right)$$

(2) $$R_{1,F}\left(\omega_{F}\right)=R_{1,F}^{intra}\left(\omega_{F}\right)+R_{1,F}^{inter,FF}\left(\omega_{F}\right)+R_{1,F}^{inter,FH}\left(\omega_{F}\right)$$

The intra-ionic relaxation contributions, $R_{1,H}^{intra}\left(\omega_{H}\right)$ and $R_{1,F}^{intra}\left(\omega_{F}\right)$, for the cation and the anion, respectively, originate from ^1^H–^1^H dipole–dipole interactions within the cation ($R_{1,H}^{intra}\left(\omega_{H}\right))$ and ^19^F–^19^F dipole–dipole interactions within the anion ($R_{1,F}^{intra}\left(\omega_{F}\right)$). These interactions fluctuate in time as a result of rotational dynamics of the ions. Consequently, the relaxation contributions $R_{1,H}^{intra}\left(\omega_{H}\right)$ and $R_{1,F}^{intra}\left(\omega_{F}\right)$ can be expressed as [6,8,9,12,13,21]:

(3) $$R_{1,H}^{intra}\left(\omega_{H}\right)=C_{DD}^{HH}\left[\frac{\tau_{rot}^{C}}{1+{\left(\omega_{H}\tau_{rot}^{C}\right)}^{2}}+\frac{4\tau_{rot}^{C}}{1+{\left(2\omega_{H}\tau_{rot}^{C}\right)}^{2}}\right]$$

(4) $$R_{1,F}^{intra}\left(\omega_{F}\right)=C_{DD}^{FF}\left[\frac{\tau_{rot}^{A}}{1+{\left(\omega_{F}\tau_{rot}^{A}\right)}^{2}}+\frac{4\tau_{rot}^{A}}{1+{\left(2\omega_{F}\tau_{rot}^{A}\right)}^{2}}\right]$$

where the parameters $\tau_{rot}^{C}$ and $\tau_{rot}^{A}$ denote the rotational correlation times of the cation and the anion, respectively, while $C_{DD}^{HH}$ and $C_{DD}^{FF}$ denote the corresponding dipolar relaxation constants determined by the structure of the ions. The inter-ionic dipole–dipole couplings fluctuate in time due to the relative translation diffusion of the interaction ions. The relaxation contributions $R_{1,H}^{inter,HH}\left(\omega_{H}\right)$ and $R_{1,F}^{inter,FF}\left(\omega_{F}\right)$ originate from ^1^H–^1^H (cation–cation) and ^19^F–^19^F (anion–anion) interactions and can be expressed as [6,9,12,21,31,32]:

(5) $$R_{1,H}^{inter,\;HH}\left(\omega_{H}\right)=\frac{108}{5}{\left(\frac{\mu_{0}}{4\pi }\gamma_{H}^{2}\hbar \right)}^{2}\frac{1}{d_{CC}^{3}}N_{H}\times \;\int_{0}^{\infty }\frac{u^{4}}{81+9u^{2}-2u^{4}+u^{6}}\left[\frac{\tau_{trans}^{C}}{u^{4}+{\left(\omega_{H}\tau_{trans}^{C}\right)}^{2}}+\frac{4\tau_{trans}^{C}}{u^{4}+{\left(2\omega_{H}\tau_{trans}^{C}\right)}^{2}}\right]du$$

(6) $$R_{1,F}^{inter,\;FF}\left(\omega_{F}\right)=\frac{108}{5}{\left(\frac{\mu_{0}}{4\pi }\gamma_{F}^{2}\hbar \right)}^{2}\frac{1}{d_{AA}^{3}}N_{F}\times \;\int_{0}^{\infty }\frac{u^{4}}{81+9u^{2}-2u^{4}+u^{6}}\left[\frac{\tau_{trans}^{A}}{u^{4}+{\left(\omega_{F}\tau_{trans}^{A}\right)}^{2}}+\frac{4\tau_{trans}^{A}}{u^{4}+{\left(2\omega_{F}\tau_{trans}^{A}\right)}^{2}}\right]du$$

The translational correlation times for the cation and the anion, $\tau_{trans}^{C}$ and $\tau_{trans}^{A}$, respectively are defined as: $\tau_{trans}^{C}=\frac{d_{CC}^{2}}{2D_{trans}^{C}}$ and $\tau_{trans}^{A}=\frac{d_{AA}^{2}}{2D_{trans}^{A}}$, where $d_{CC}$ and $d_{AA}$ denote the distances of the closest approach for a pair of cations and a pair of anions, respectively, while $D_{trans}^{C}$ and $D_{trans}^{A}$ are translation diffusion coefficients of the cation and of the anion. The quantities $N_{H}$ and $N_{F}$ denote the numbers of ^1^H and ^19^F nuclei per unit volume, respectively. They can be obtained from the relationship: $N_{H}=\frac{n_{H}N_{A}\varrho }{M}$ and $N_{F}=\frac{n_{F}N_{A}\varrho }{M}$, where $n_{H}$ and $n_{F}$ denote the number of hydrogen atoms per cation and the number of fluorine atoms per anion, respectively, $N_{A}$ is the Avogadro number, ϱ denotes density of the ionic liquid, while M is its molecular mass; $\gamma_{H}$ and $\gamma_{F}$ are ^1^H and ^19^F gyromagnetic factors, other symbols have their obvious meaning. Equations (1) and (2) clearly show that the ^1^H and ^19^F relaxation processes are not independent, and in this sense, they are both affected by the ^1^H–^19^F (cation–anion) interactions. The corresponding relaxation contributions can be expressed as [6,12]:

(7) $$R_{1,H}^{inter,\;HF}\left(\omega_{H}\right)=\;\frac{36}{5}{\left(\frac{\mu_{0}}{4\pi }\gamma_{H}\gamma_{F}\hbar \right)}^{2}\frac{1}{d_{CA}^{3}}N_{F}\int_{0}^{\infty }\frac{u^{4}}{81+9u^{2}-2u^{4}+u^{6}}\left[\frac{\tau_{trans}^{CA}}{u^{4}+{\left(\left(\omega_{H}-\omega_{F}\right)\tau_{trans}^{CA}\right)}^{2}}\;+\frac{3\tau_{trans}^{CA}}{u^{4}+{\left(\omega_{H}\tau_{trans}^{CA}\right)}^{2}}+\frac{6\tau_{trans}^{CA}}{u^{4}+{\left(\left(\omega_{H}+\omega_{F}\right)\tau_{trans}^{CA}\right)}^{2}}\right]$$

and

(8) $$\begin{array}{l} R_{1,F}^{inter,FH}\left(\omega_{F}\right)\\ =\frac{36}{5}{\left(\frac{\mu_{0}}{4\pi }\gamma_{H}\gamma_{F}\hbar \right)}^{2}\frac{1}{d_{CA}^{3}}N_{H}\int_{0}^{\infty }\frac{u^{4}}{81+9u^{2}-2u^{4}+u^{6}}\\ \left[\frac{\tau_{trans}^{CA}}{u^{4}+{\left(\left(\omega_{H}-\omega_{F}\right)\tau_{trans}^{CA}\right)}^{2}}+\frac{3\tau_{trans}^{CA}}{u^{4}+{\left(\omega_{F}\tau_{trans}^{CA}\right)}^{2}}+\frac{6\tau_{trans}^{CA}}{u^{4}+{\left(\left(\omega_{H}+\omega_{F}\right)\tau_{trans}^{CA}\right)}^{2}}\right] \end{array}$$

where $d_{CA}$ denotes the distance of THE closest approach between the cation and the anion, while $\tau_{trans}^{CA}=\frac{d_{CA}^{2}}{D_{trans}^{CA}}$. The diffusion coefficient $D_{trans}^{CA}$ describes the relative translation diffusion of the cation and the anion. In case the translation movement of these ions is uncorrelated, one gets:$\;D_{trans}^{CA}=D_{trans}^{C}+D_{trans}^{A}$.

## 3. Results and Analysis

**Figure 1:**
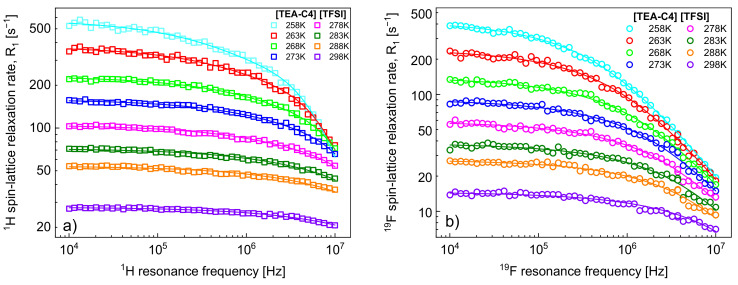
(**a**) ^1^H and (**b**) ^19^F spin-lattice relaxation data for [TEA-C4][TFSI]; solid lines—fits in terms of the model of Section 2.

^1^H and ^19^F spin-lattice relaxation data for [TEA-C4][TFSI] are shown in Figure 1a,b, respectively. The figures include fits performed in terms of the model outlined in Section 2.

It has turned out that the ^1^H spin-lattice relaxation data can be reproduced without the relaxation contribution, $R_{1,H}^{inter,\;HF}\left(\omega_{H}\right)$, associated with the cation–anion, ^1^H–^19^F, dipole–dipole interactions. Consequently, the fits of the ^1^H relaxation data include four adjustable parameters: $D_{trans}^{C}$, $d_{CC}$, $C_{DD}^{HH}$ and $\tau_{rot}^{C}$. The number of ^1^H nuclei per unit volume, $N_{H}$, has been calculated as described in Section 2; for [TEA-C4][TFSI] (C_12_H_24_F_6_N_2_O_2_S_2_) one gets: M = 438.45 g/mol, ϱ = 1.332 g/mol, $n_{H}$ = 24; consequently $N_{H}$ = 4.39·10^28^/m^3^. The parameters are collected in Table 1.

**Figure 2:**
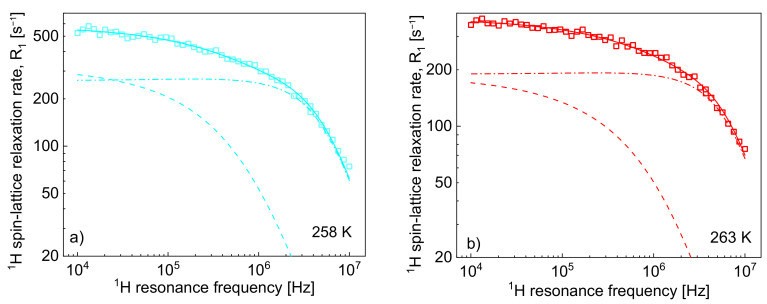

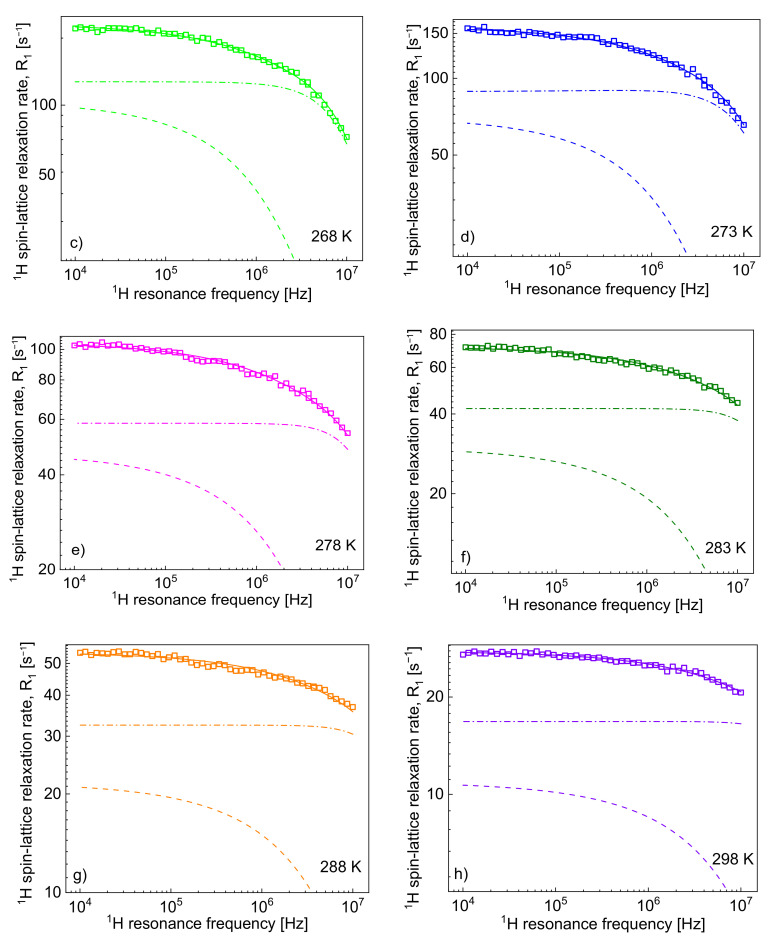
^1^H spin-lattice relaxation rates, $R_{1,H}\left(\omega_{H}\right)$, for [TEA-C4][TFSI]; solid lines—theoretical fits decomposed into $R_{1,H}^{intra}\left(\omega_{H}\right)$ (dashed-dotted lines) and $R_{1,H}^{inter,HH}\left(\omega_{H}\right)$ (dashed lines) at different temperatures (**a**–**h**).

Taking into account that $n_{F}$ = 6 and, hence, $N_{F}$ = 1.10·10^28^/m^3^ ($N_{F}$/$N_{H}$ = 1/4), the $R_{1,H}^{inter,\;HF}\left(\omega_{H}\right)$ relaxation contribution to the $R_{1,H}\left(\omega_{H}\right)$ relaxation rates can indeed be small; however, the contribution also depends on other factors—we shall come back to this subject later. Figure 2 shows the ^1^H spin-lattice relaxation rates, $R_{1,H}\left(\omega_{H}\right)$, decomposed into the individual relaxation contributions: $R_{1,H}^{intra}\left(\omega_{H}\right)$ and $R_{1,H}^{inter,\;HF}\left(\omega_{H}\right)$.

**Figure 3:**
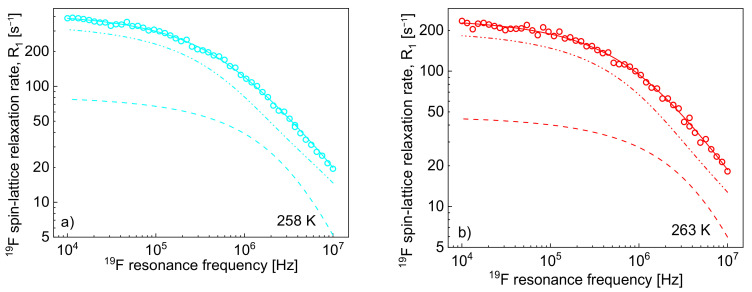

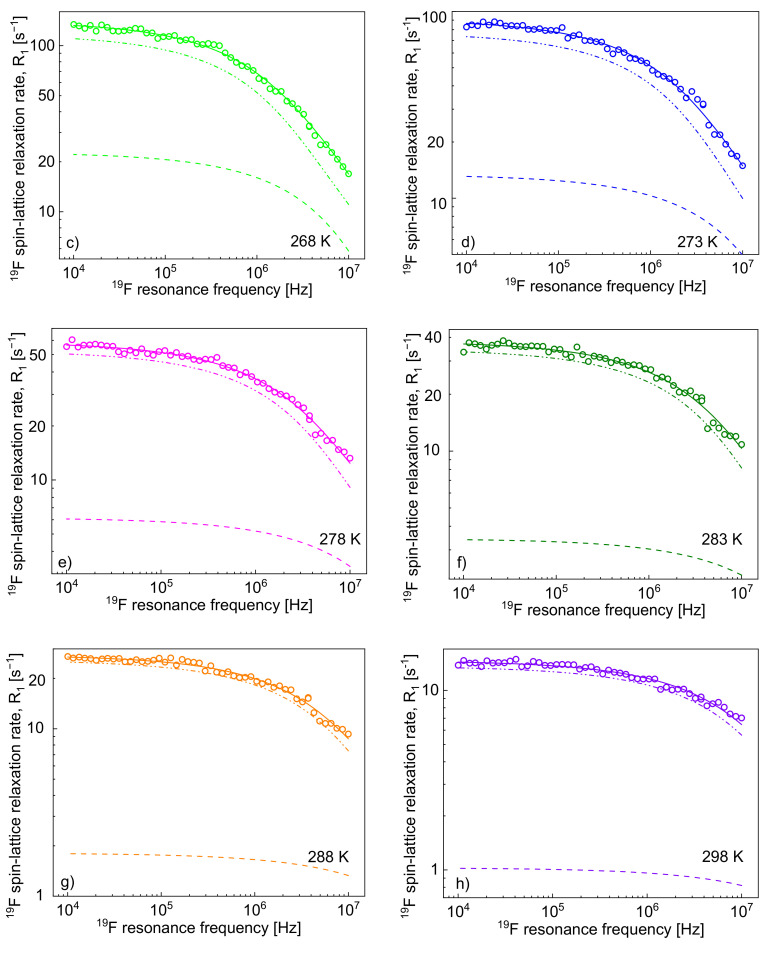
^19^F spin-lattice relaxation rates, $R_{1,F}\left(\omega_{F}\right)$, for [TEA-C4][TFSI]; solid lines—theoretical fits decomposed into $R_{1,F}^{inter,FF}\left(\omega_{F}\right)$ (dashed lines) and $R_{1,F}^{inter,FH}\left(\omega_{F}\right)$ (dashed-dotted-dotted lines) at different temperatures (**a**–**h**).

One can clearly see from the decomposition that when the rotational correlation time, $\tau_{rot}^{C}$, becomes of the order of 5·10^−9^ s (or shorter), the relaxation contribution $R_{1,H}^{intra}\left(\omega_{H}\right)$ becomes frequency independent (as then the condition $\omega_{H}\tau_{rot}^{C}\ll 1$ is approached). However, as the dipolar relaxation constant $C_{DD}^{HH}$ has unambiguously been determined from the analysis of the relaxation data at lower temperatures and kept unchanged with temperature, the values of $\tau_{rot}^{C}$ can be determined even when the $R_{1,H}^{intra}\left(\omega_{H}\right)$ is frequency independent. In Table 2, the ratio $\tau_{trans}^{C}/\tau_{rot}^{C}$ has been calculated. The value monotonically decreases with temperature from 13.3 to 7.0. Following this line, the ^19^F spin-lattice relaxation data have been analysed in terms of the model presented in Section 2 (Figure 3). In this case, the ^1^H–^19^F relaxation term, $R_{1,F}^{inter,FH}\left(\omega_{F}\right)$, gives a considerable contribution to the overall ^19^F spin-lattice relaxation rates, $R_{1,F}\left(\omega_{F}\right)$. This is not surprising considering that $N_{H}$/$N_{F}$ = 4 and $R_{1,F}^{inter,FH}\left(\omega_{F}\right)$ is proportional to $N_{H}$ (Equation (8)). However, as a result of fast rotational dynamics of TFSI anions and, presumably, a relatively small dipolar relaxation constant ($C_{DD}^{FF})$, the relaxation contribution $R_{1,F}^{intra}\left(\omega_{F}\right)$ has turned out to be negligible. The obtained parameters are collected in Table 2.

The decomposition evidently shows that the cation–anion, ^1^H–^19^F, relaxation contribution, $R_{1,F}^{inter,FH}\left(\omega_{F}\right)$ dominates the ^19^F spin-lattice relaxation rate, $R_{1,F}\left(\omega_{F}\right)$, and its importance increases with increasing temperature.

**Figure 4:**
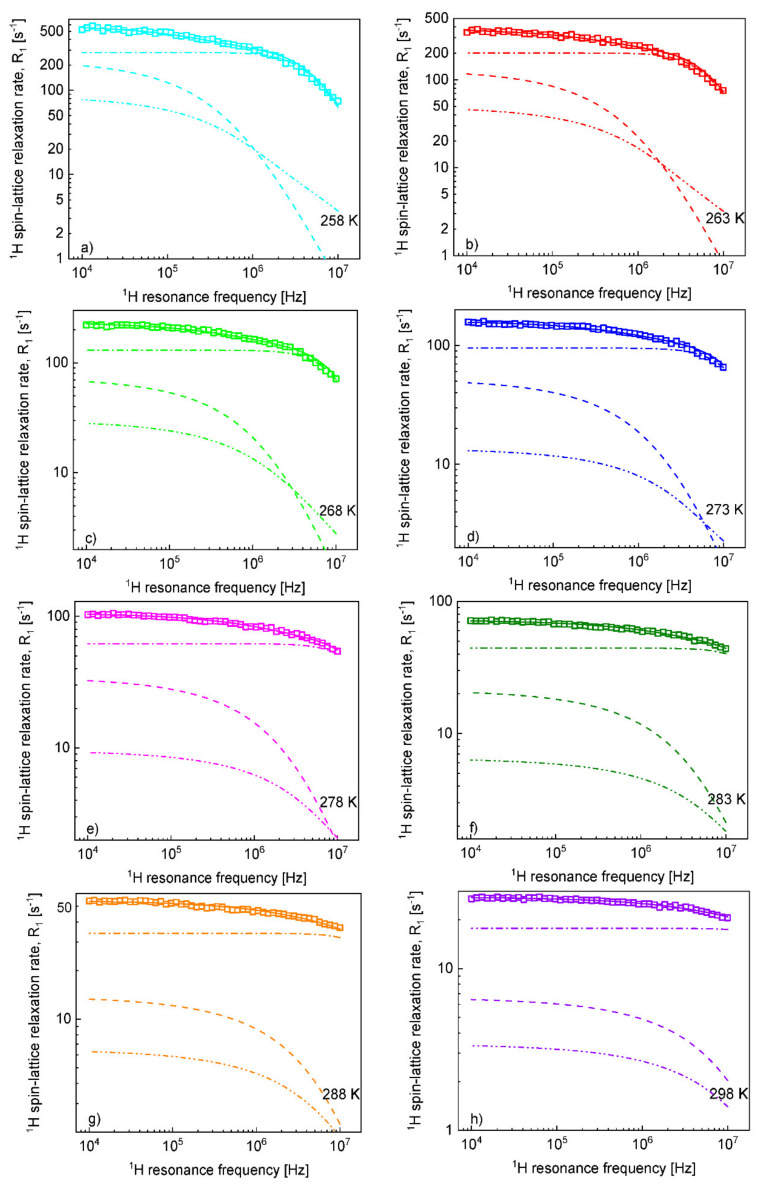
^1^H spin-lattice relaxation rates, $R_{1,H}\left(\omega_{H}\right)$, for [TEA-C4][TFSI]; solid lines—theoretical fits decomposed into $R_{1,H}^{inter,HH}\left(\omega_{H}\right)$ (dashed lines), $R_{1,H}^{inter,HF}\left(\omega_{H}\right)$ (dashed-dotted-dotted lines) and $R_{1,H}^{intra}\left(\omega_{H}\right)$ (dashed-dotted lines) at different temperatures (**a**–**h**).

Knowing the relative cation–anion translation diffusion coefficient, $D_{trans}^{CA}$, and the distance of the closest approach, $d_{CA}$, one can verify the importance of the $R_{1,F}^{inter,HF}\left(\omega_{F}\right)$ relaxation contribution. For this purpose, the ^1^H spin-lattice relaxation data have been fitted again, including the $R_{1,F}^{inter,HF}\left(\omega_{F}\right)$ relaxation contribution with $D_{trans}^{CA}$ and $d_{CA}$ fixed to the values obtained from the analysis of the ^19^F spin-lattice relaxation data (Figure 4).

The obtained parameters are collected in Table 3. The diffusion coefficients have been compared with values obtained by means of NMR gradient methods [34]; at low temperatures, the diffusion is too slow for applying NMR gradient diffusometry.

**Figure 5:**
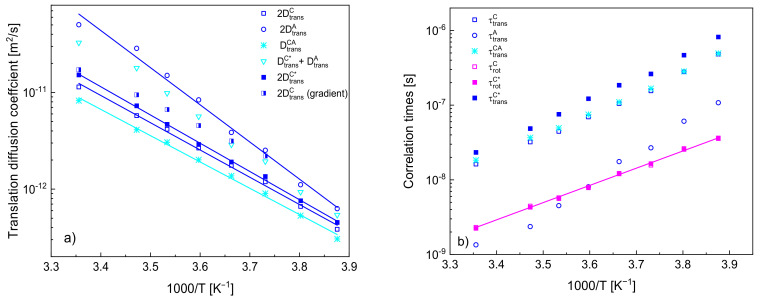
(**a**) Translation diffusion coefficients for [TEA-C4] cation and [TFSI] anion, solid lines—fits according to the Arrhenius law; (**b**) translational and rotational correlation times with a corresponding fit of the Arrhenius law.

In Figure 5a, the obtained diffusion coefficients are plotted versus reciprocal temperature. For comparison, the translation diffusion coefficients of the cation and of the anion, $D_{trans}^{C}$, $D_{trans}^{C*}$ and $D_{trans}^{A}$, have been multiplied by a factor of two to account for the relative translation motion. The values of the diffusion coefficients for the cation are compared with those obtained from NMR gradient diffusometry [34]. Figure 5b includes translational and rotational correlation times (the first one calculated from the diffusion coefficients and the distances of the closest approach).

The quantities show linear dependencies on reciprocal temperature according to the Arrhenius law: $D_{trans}\left(T\right)=D_{trans,0}exp\left(-\frac{E_{A}}{RT}\right)$ and $\tau_{c}\left(T\right)=\tau_{c,0}exp\left(\frac{E_{A}}{RT}\right)$, where $D_{trans}$ and $\tau_{c}$ denote a translation diffusion coefficient and a correlation time, respectively, $D_{trans,0}$ and $\tau_{c,0}$ are the high temperature limits, $E_{A}$ denotes an activation energy while R is the gas constant. The activation energies for $D_{trans}^{C}$, $D_{trans}^{C*}$, $D_{trans}^{A}$ and $D_{trans}^{CA}$ yield: (23.4 ± 0.6) kJ/(mol·K), (24.3 ± 0.4) kJ/(mol·K), (31.9 ± 1.2) kJ/(mol·K) and (22.6 ± 0.5) kJ/(mol·K), respectively, while the activation energy for $\tau_{rot}^{C*}$ yields (19.4 ± 0.3) kJ/(mol·K).

## 4. Discussion

The ^1^H and ^19^F spin-lattice relaxation experiments have been performed in the temperature range from 258 to 298 K. As the melting point of [TEA-C4][TFSI] is 289.1 K, the data have been collected (except at the highest temperature of 298K) in the supercooled state. In the first step, the ^1^H spin-lattice relaxation data have been analysed considering only ^1^H–^1^H dipole–dipole interactions, i.e., neglecting the cation–anion ^1^H–^19^F dipole–dipole coupling. The reason for neglecting the relaxation contribution associated with the ^1^H–^19^F dipole–dipole interactions is $N_{F}$ being four times smaller than $N_{H}$. The attempt has turned out to be successful yielding the values of the translation diffusion coefficient for the cation from 1.92·10^−13^ m^2^/s at 258 K to 5.70·10^−12^ m^2^/s at 298 K. Combining these values with the cation–cation distance of the closest approach of 4.30 Å, for the translational correlation, the range from 4.82·10^−7^ s (258 K) to 1.62·10^−8^ s (298 K) has been obtained. The analysis has also revealed the rotational correlation time for the cation—it ranges from 3.63·10^−8^ s at 258 K to 2.31·10^−9^ s at 298 K. Notably, the ratio $\tau_{trans}^{C}/\tau_{rot}^{C}$ decreases monotonically from 13.3 at 258 K to 7.0 at 298 K. The Stokes equation predicts for spherical molecules the ratio between the translational and the rotational correlation times equal to 9 [27], while for “real” molecular liquids, values in the range of 20–40 have been obtained [35].

As far as the ^19^F relaxation data are concerned, the relaxation contribution associated with the cation–anion ^1^H–^19^F dipole–dipole interactions (mediated by the cation–anion relative translational diffusion) is not only non-negligible, but it dominates the relaxation contribution caused by the anion–anion ^19^F–^19^F interactions, as shown in Figure 3. The diffusion coefficient for the anion ranges from 3.14·10^−13^ m^2^/s at 258 K and 2.51·10^−11^ m^2^/s at 298 K, which means that the anions diffuse faster than the cations. With the anion–anion distance of the closest approach, 2.60 Å, the translational correlation time for the anion ranges from 1.08·10^−7^ s at 258 K to 1.35·10^−9^ s at 298 K. At the same time, the relative cation–anion translation diffusion coefficient ranges from 3.05·10^−13^ m^2^/s at 258 K to 7.76·10^−12^ m^2^/s at 298 K, which gives (for the cation–anion distance of the closet approach of 3.88 Å) the range of the corresponding correlation times from 4.94·10^−7^ to 1.83·10^−8^ s. Due to fast rotation of the anions, the relaxation contribution associated with the intra-anionic ^19^F–^19^F interactions turned out to be negligible.

Knowing the cation–anion diffusion coefficients and the distance of the closest approach for the ions, the ^1^H spin-lattice relaxation data have been analysed again, accounting for the (known) ^1^H–^19^F relaxation contribution. The analysis has led to somewhat larger translation diffusion coefficients of the cation and a considerably larger value of the cation–cation distance of the closest approach, 5.95 Å. Consequently, the translational correlation time has become longer (from 8.19·10^−7^ s at 258 K to 2.33·10^−8^ s at 298 K). Taking into account that the rotational correlation time has only slightly been affected by the extended relaxation scenario, the ratio between the correlation times has become larger, yielding 23.0 at 258 K and monotonically decreasing to 10.4 at 298 K. As one can see in Figure 4, the ^1^H–^19^F relaxation contribution to the ^1^H relaxation has turned out to be of importance.

In Figure 5a, the obtained translation diffusion coefficients have been compared versus reciprocal temperature. The comparison clearly shows that (as already pointed out) the translation diffusion of the anions is faster than that of the cations. The ratio between the diffusion coefficients of the anion and of the cation (in the case of the cation, we refer to the values obtained when the ^1^H–^19^F relaxation contribution is accounted for) yields from 3.30 at 298 K to 1.45 at 258 K—this indicates that the diffusion coefficients tend to converge at low temperatures. In order to compare the relative cation–anion diffusion coefficients with the cation and anion diffusion coefficients, the last two values have been multiplied by two in Figure 5; moreover, a sum of the diffusion coefficients of the action and of the anion has been plotted. One can clearly see from the comparison that the relative cation–anion translation diffusion coefficients are smaller than the sum—the ratio yields from 0.40 at 258 K to 0.56 at 298 K.

Eventually, it is worth comparing the translation diffusion coefficients of the cations obtained from the analysis with those obtained from NMR diffusometry [34]. The values are in good agreement at the high temperature (298 K), but they progressively deviate from each other with decreasing temperature—the value obtained from the diffusometry measurements is larger, and the ratio yields 1.62 at 263 K. In this context, one should take into account that the diffusion is slow from the perspective of NMR diffusometry, and the measurements at low temperatures pose a challenge (one of the reasons is a short spin-spin relaxation time). Nevertheless, the discrepancies systematically increase, and this brings one to the point when one should consider the subject of correlation in the translation diffusion of cations. In analogy to the cation–anion relative translation diffusion coefficient, the output of the analysis of the ^1^H spin-lattice relaxation data is, in fact, the relative cation–cation translation diffusion coefficient that has been treated as equal to twice the self-diffusion coefficient $D_{trans}^{C*}$ (or $D_{trans}^{C}$). However, with decreasing temperature, the values of $D_{trans}^{C*}$ (being, in fact equal to a half of the relative translation diffusion coefficient) become progressively smaller compared to those obtained by means of NMR diffusometry, suggesting that the relative cation–cation translation movement becomes more correlated. This might suggest that translation movement of one cation triggers (to some extend) a displacement of neighbouring cations in the direction of the first one (consequently, their relative diffusion becomes slower as their distance changes less in time than in the case of uncorrelated dynamics).

It has turned out that all dynamical processes follow the Arrhenius law with the activation energies for the translation diffusion of the cations and for the relative cation–anion motion being similar (23.4 kJ/(mol·K) and 22.6 kJ/(mol·K), respectively), while the activation energy for the anion diffusion is higher (31.9 kJ/(mol·K)). The activation energy for the rotational dynamic of the cation (19.4 kJ/(mol·K)) is somewhat lower than for the translation diffusion.

As already pointed out, correlated dynamics of ions is considered as an important factor enhancing ionic conductivity and, consequently, determining the applicability of some ionic liquids as electrolytes. These kinds of studies offer the possibility of obtaining a direct insight into correlation effects in translational dynamics of ions and, hence, open the possibility to verify this hypothesis. Moreover, from the perspective of fundamental studies, this is a way to enquire into the relationship between structural properties of ionic liquids and correlated dynamics of the ions.

## 5. Materials and Methods

^1^H and ^19^F spin-lattice relaxation measurements have been performed for [TEA-C4][TFSI] in the frequency range 10 kHz to 10 MHz (referring to the ^1^H resonance frequency) versus temperature, from 258 to 298 K using an NMR relaxometer, produced by Stelar s.r.l. (Mede (PV), Italy). The temperature was controlled with an accuracy of 0.5 K. The experiments started at a higher temperature, which was progressively decreased. For each resonance frequency, 32 magnetisation values versus time in a logarithmic time scale have been recorded. Below 4 MHz, pre-polarisation at 0.19 T was applied. The switching time of the magnet was set to 3 ms.

The relaxation processes (^1^H and ^19^F) turned out to be single-exponential for all measured temperatures in the whole frequency range, as shown in the Supplementary Materials (Figures S1–S8 for ^1^H and Figures S9–S16 for ^19^F).

[TEA-C4][TFSI] was obtained according to the protocols described in References [34,36]. Quaternary tetrabutyltriethylammonium bromide was subjected to metathesis reaction with lithium bis(trifluoromethanesulfonyl)imide, purified and dried. The melting point of [TEA-C4][TFSI] was established at 289.1 K [34].

## 6. Conclusions

A thorough analysis of ^1^H and ^19^F spin-lattice relaxation data for [TEA-C4][TFSI] collected at a broad range of resonance frequencies (from about 10 kHz to 10 MHz) and a temperature range from 258 to 298 K has revealed relative (cation-cation, anion–anion and cation–anion) translation diffusion coefficients. A comparison of the cation–cation diffusion coefficients with corresponding values obtained by means of NMR gradient diffusometry indicates correlation effects in the relative cation–cation translation movement that become more pronounced with decreasing temperature (at 263 K, the ratio between the self-diffusion coefficient of the cation obtained by means of NMR diffusometry and a half of the relative cation–cation translation diffusion coefficient reaches about 1.6, while at 298 K, the two quantities are very similar). It has also turned out that the translation motion of the anions is faster than that of the cations (the ratio between the relative anion–anion and cation–cation translation diffusion coefficients ranges from 3.30 at 298 K to 1.45 at 258 K), although with decreasing temperature, the values tend to converge. At the same time, the relative cation–anion translation diffusion coefficients are smaller than the sum of the diffusion coefficients of the cation and of the anion, indicating a correlated cation–anion movement. The analysis has also allowed determining the rotational correlation time of the action. The ratio between the translational and rotational correlation times monotonically decreases with increasing temperature and lies in the range from about 23 to about 10, similarly to molecular liquids.

Eventually, one should point out that the presented results show the unique potential of NMR relaxometry to reveal dynamical properties of ionic liquids and (very importantly) shed light on correlation effects in the translational dynamics.

## Figures and Tables

**Table 1:** Parameters characterising the translational and rotational dynamics of the TEA-C4 cation in [TEA-C4][TFSI]; $C_{DD}^{HH}$ = 1.49·10^9^ Hz^2^, $d_{CC}$ = 4.30 Å. The correlation time $\tau_{trans}^{C}$ has been calculated from the relationship:$\;\tau_{trans}^{C}=\frac{d_{CC}^{2}}{2D_{trans}^{C}}$.

Temp. (K)	$D_{trans}^{C}\;(m^{2}/s)$	$\tau_{rot}^{C}\;(s)$	Rel. Error (%)	$\tau_{trans}^{C}\;(s)$	$\tau_{trans}^{C}/\tau_{rot}^{C}$
258	1.92·10^−13^	3.63·10^−8^	10.7	4.82·10^−7^	13.3
263	3.31·10^−13^	2.62·10^−8^	8.0	2.79·10^−7^	10.6
268	5.95·10^−13^	1.57·10^−8^	3.1	1.55·10^−7^	9.9
273	8.80·10^−13^	1.22·10^−8^	2.9	1.05·10^−7^	8.6
278	1.33·10^−12^	7.99·10^−9^	2.6	6.95·10^−8^	8.7
283	2.08·10^−12^	5.75·10^−9^	2.9	4.44·10^−8^	7.7
288	2.88·10^−12^	4.45·10^−9^	3.4	3.21·10^−8^	7.2
298	5.70·10^−12^	2.31·10^−9^	1.2	1.62·10^−8^	7.0

**Table 2:** Parameters characterising the translational and rotational dynamics of TEA-C4 anion in [TEA-C4][TFSI]; $C_{DD}^{HH}$ = 1.49·10^9^ Hz^2^, $d_{CC}$ = 4.30 Å. The correlation time $\tau_{trans}^{C}$ has been calculated from the relationship:$\;\tau_{trans}^{C}=\frac{d_{CC}^{2}}{2D_{trans}^{C}}$.

Temp. (K)	$D_{trans}^{A}\;(m^{2}/s)$	$D_{trans}^{CA}\;(m^{2}/s)$	Rel. Error (%)	$\tau_{trans}^{A}\;(s)$	$\tau_{trans}^{CA}\;(s)$
258	3.14·10^−13^	3.05·10^−13^	7.5	1.08·10^−7^	4.94·10^−7^
263	5.55·10^−13^	5.32·10^−13^	18.0	6.09·10^−8^	2.83·10^−7^
268	1.26·10^−12^	9.02·10^−13^	10.9	2.68·10^−8^	1.67·10^−7^
273	1.93·10^−12^	1.37·10^−12^	11.1	1.75·10^−8^	1.10·10^−7^
278	4.19·10^−12^	2.01·10^−12^	11.6	8.07·10^−9^	7.49·10^−8^
283	7.50·10^−12^	3.05·10^−12^	16.7	4.51·10^−9^	4.94·10^−8^
288	1.43·10^−11^	4.11·10^−12^	12.0	2.36·10^−9^	3.66·10^−8^
298	2.51·10^−11^	7.76·10^−12^	9.9	1.35·10^−9^	1.83·10^−8^

**Table 3:** Parameters characterising the translational and rotational dynamics of TEA-C4 cations in [TEA-C4][TFSI] including the $R_{1,F}^{inter,HF}\left(\omega_{F}\right)$ relaxation contribution; $C_{DD}^{HH}$ = 1.62·10^9^ Hz^2^, $d_{CC}^{*}$ = 5.95 Å. The correlation time $\tau_{trans}^{C*}$ has been calculated from the relationship:$\;\tau_{trans}^{C*}=\frac{{\left(d_{CC}^{*}\right)}^{2}}{2D_{trans}^{C*}}$ (“* ” indicates the presence of the $R_{1,F}^{inter,HF}\left(\omega_{F}\right)$ relaxation contribution). The last column includes the value of the diffusion coefficients for the cation obtained by means of NMR gradient methods [34].

Temp. (K)	$D_{trans}^{C*}\;(m^{2}/s)$	$\tau_{rot}^{C*}\;(s)$	Rel. Error (%)	$\tau_{trans}^{C*}\;(s)$	$\tau_{trans}^{C*}/\tau_{rot}^{C*}$
258	2.16·10^−13^	3.56·10^−8^	9.7	8.19·10^−7^	23.0
263	3.79·10^−13^	2.55·10^−8^	7.8	4.67·10^−7^	18.3
268	6.75·10^−13^	1.65·10^−8^	3.1	2.62·10^−7^	15.9
273	9.55·10^−13^	1.20·10^−8^	2.8	1.85·10^−7^	15.4
278	1.45·10^−12^	7.83·10^−9^	2.2	1.22·10^−7^	15.5
283	2.34·10^−12^	5.61·10^−9^	2.3	7.56·10^−8^	13.5
288	3.64·10^−12^	4.29·10^−9^	2.8	4.86·10^−8^	11.3
298	7.60·10^−12^	2.24·10^−9^	1.2	2.33·10^−8^	10.4

## Supplementary Materials

The following are available online at https://www.mdpi.com/article/10.3390/ijms22179117/s1, ^1^H and ^19^F magnetization curves (magnetization versus time) for butyltriethylammonium bis(trifluoromethylsulfonyl) imide. Solid lines denote single exponential fits (Figures S1–S16).
