# Supplementary material for: Correlated Dynamics in Ionic Liquids by Means of NMR Relaxometry: Butyltriethylammonium bis(Trifluoromethanesulfonyl)imide as an Example
Source: Int J Mol Sci. 2021 Aug 24;22(17):9117. doi: 10.3390/ijms22179117 (PMC8431572; doi:10.3390/ijms22179117)
Supplement: Supplementary file 1 [file ijms-22-09117-s001.zip › ijms-1326494-supplementary.pdf]

Supplementary Material

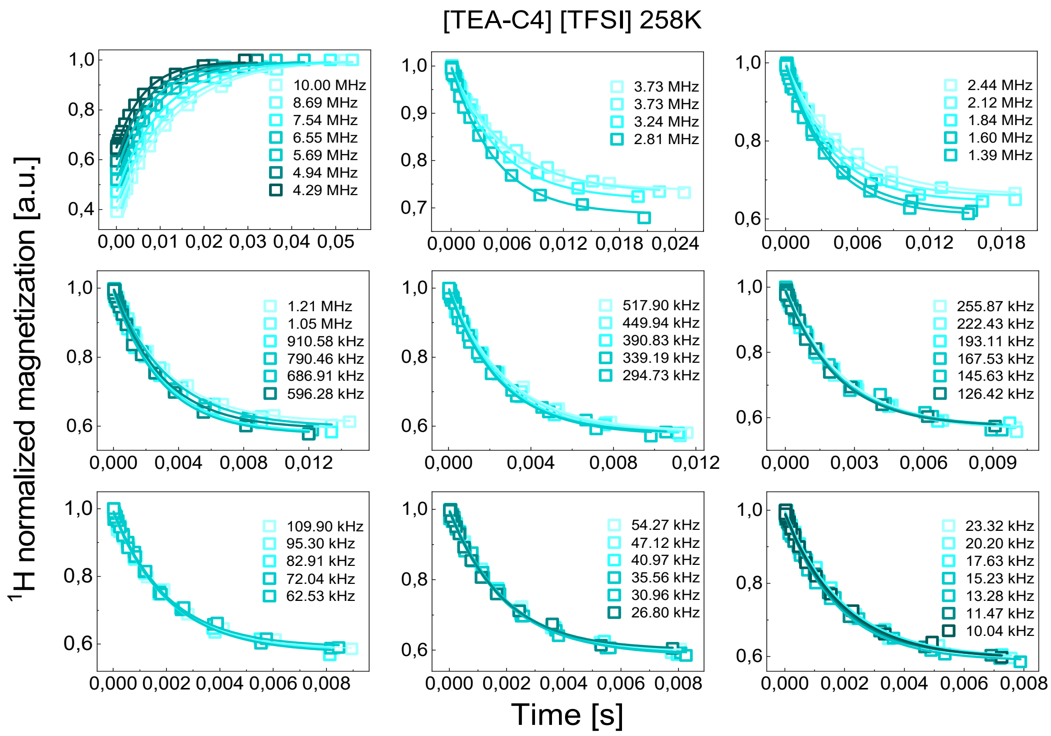

Figure S1. ^{1}H magnetization curves for [TEA-C4] [TFSI] at 258K. ^{1}H and ^{19}F magnetization curves (magnetization versus time) for butyltriethylammonium bis(trifluoromethylsulfonyl)imide. Solid lines denote single exponential fits.

[TEA-C4] [TFSI] 263 K

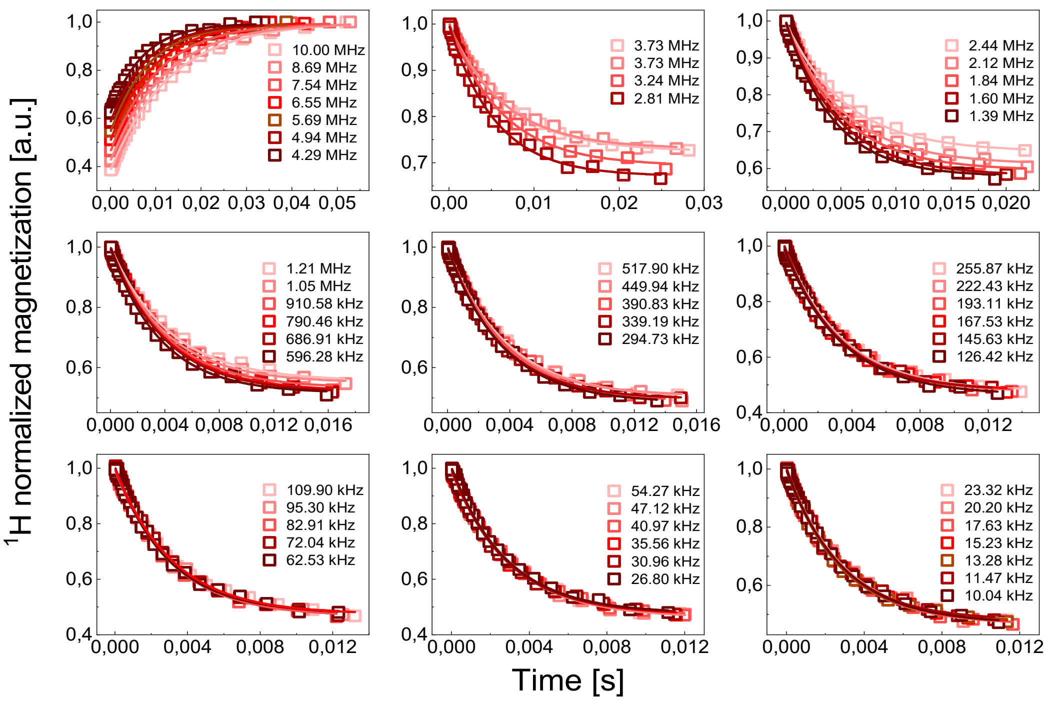

Figure S2. ^{1}H magnetization curves for [TEA-C4] [TFSI] at 263K.

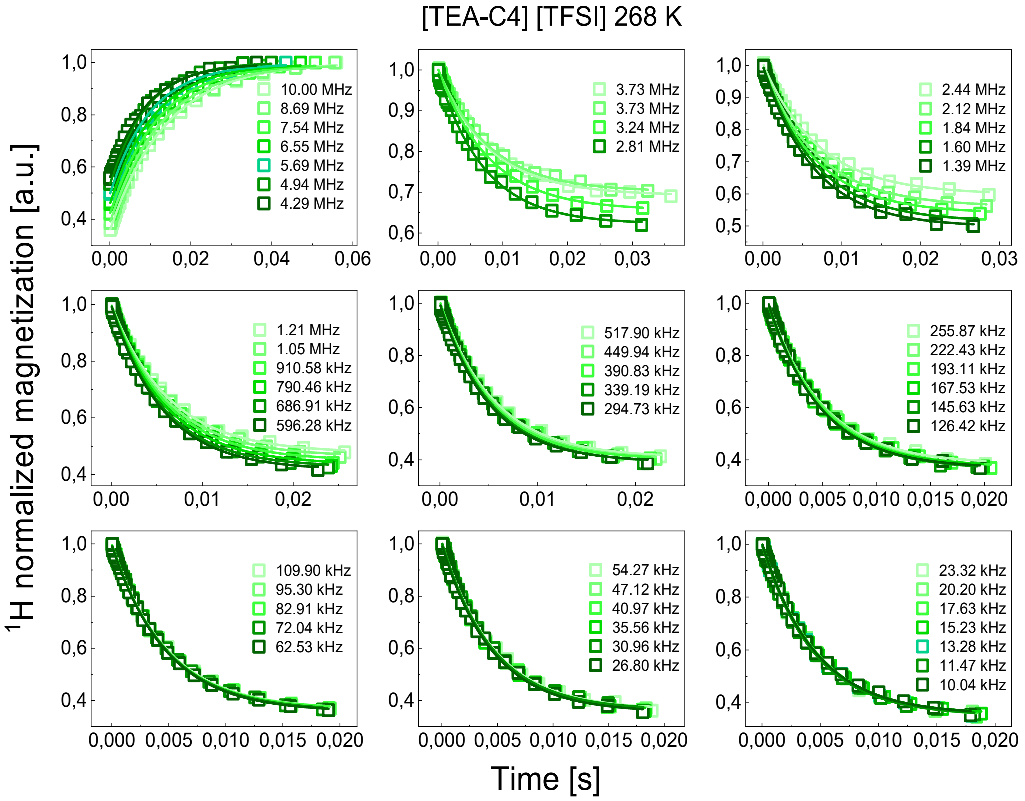

Figure S3. ^{1}H magnetization curves for [TEA-C4] [TFSI] at 268K.

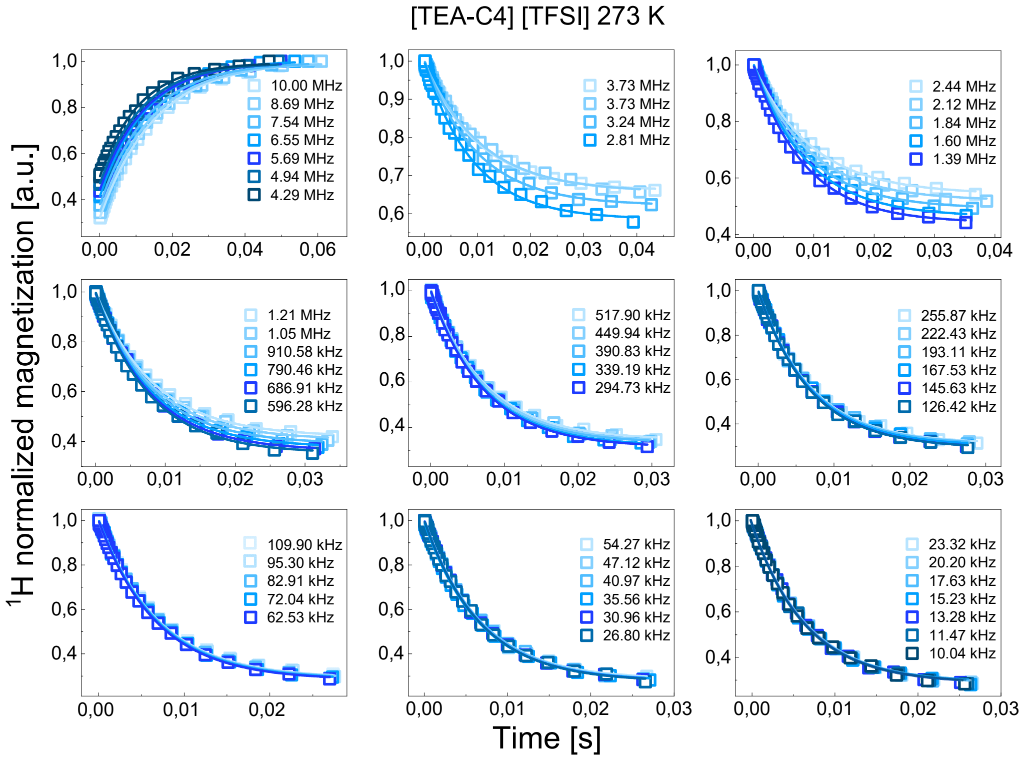

Figure S4. ^{1}H magnetization curves for [TEA-C4] [TFSI] at 273K.

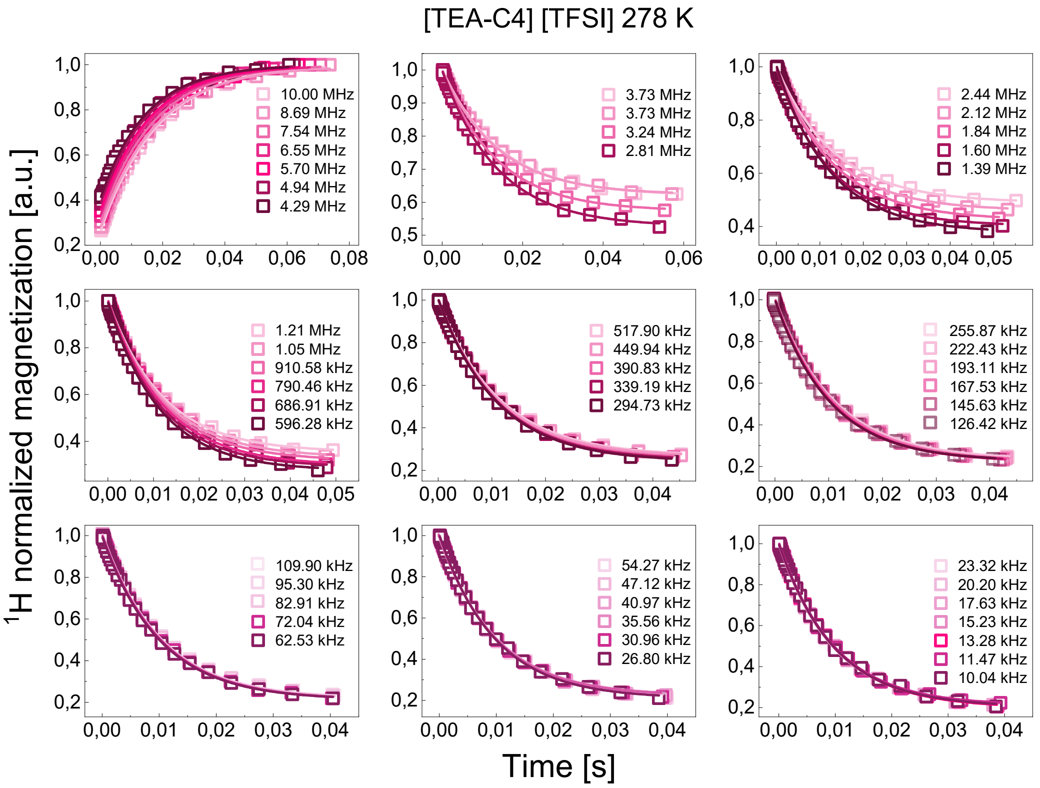

Figure S5. ^{1}H magnetization curves for [TEA-C4] [TFSI] at 278K.

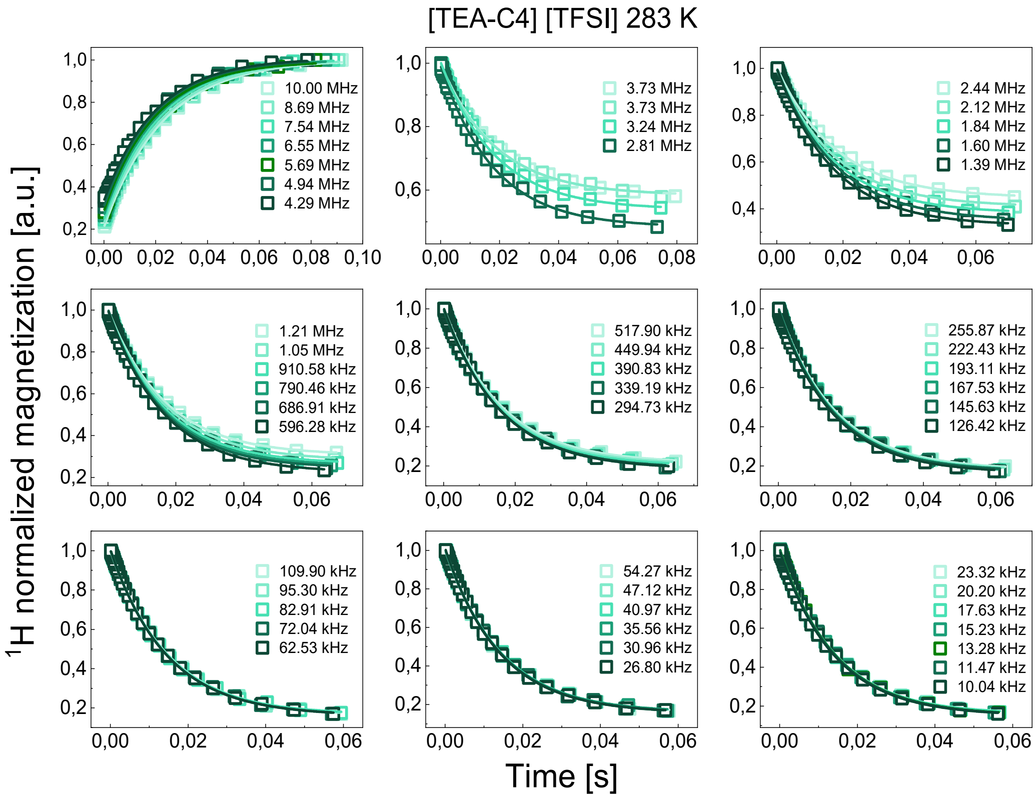

Figure S6. ^{1}H magnetization curves for [TEA-C4] [TFSI] at 283K.

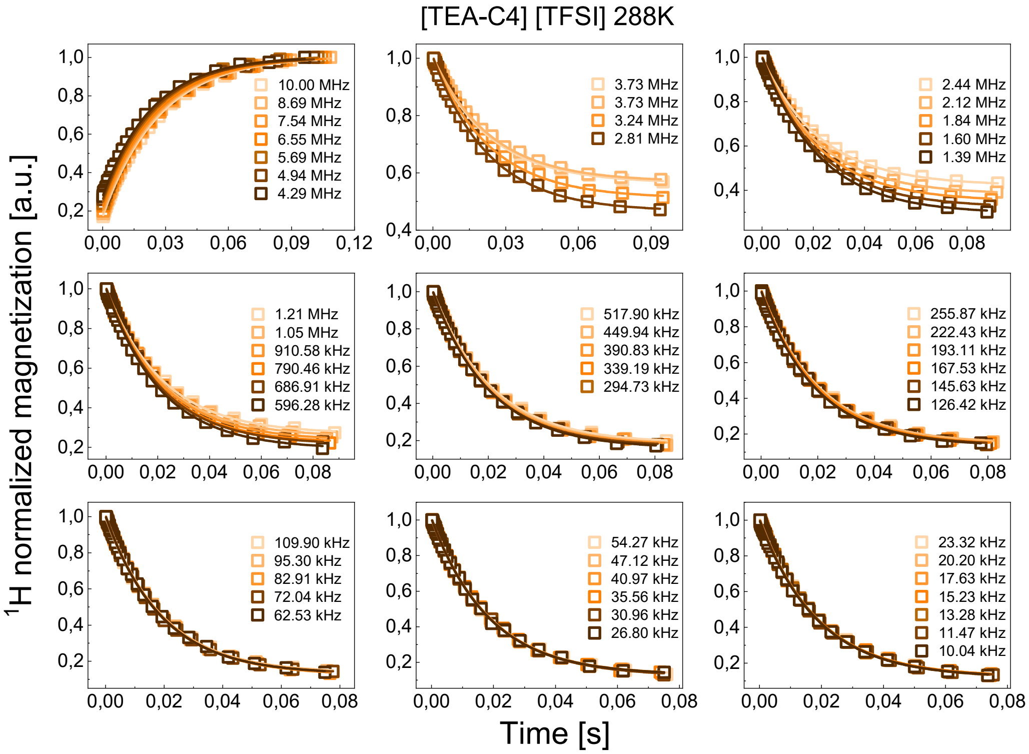

Figure S7. ^{1}H magnetization curves for [TEA-C4] [TFSI] at 288K.

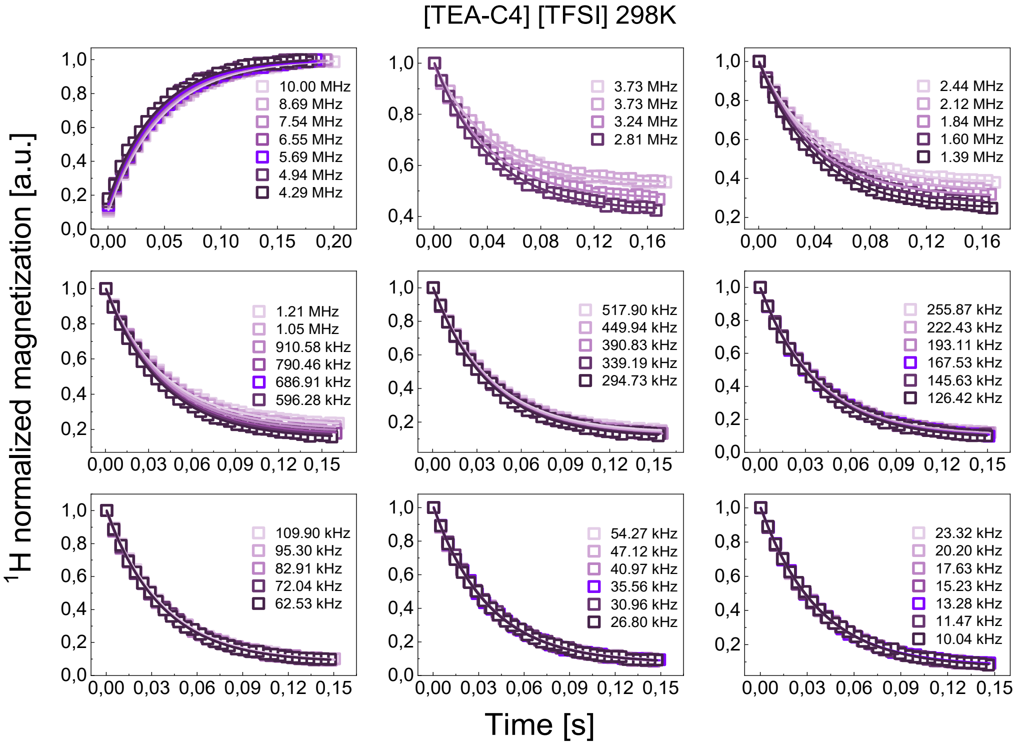

Figure S8. ^{1}H magnetization curves for [TEA-C4] [TFSI] at 298K.

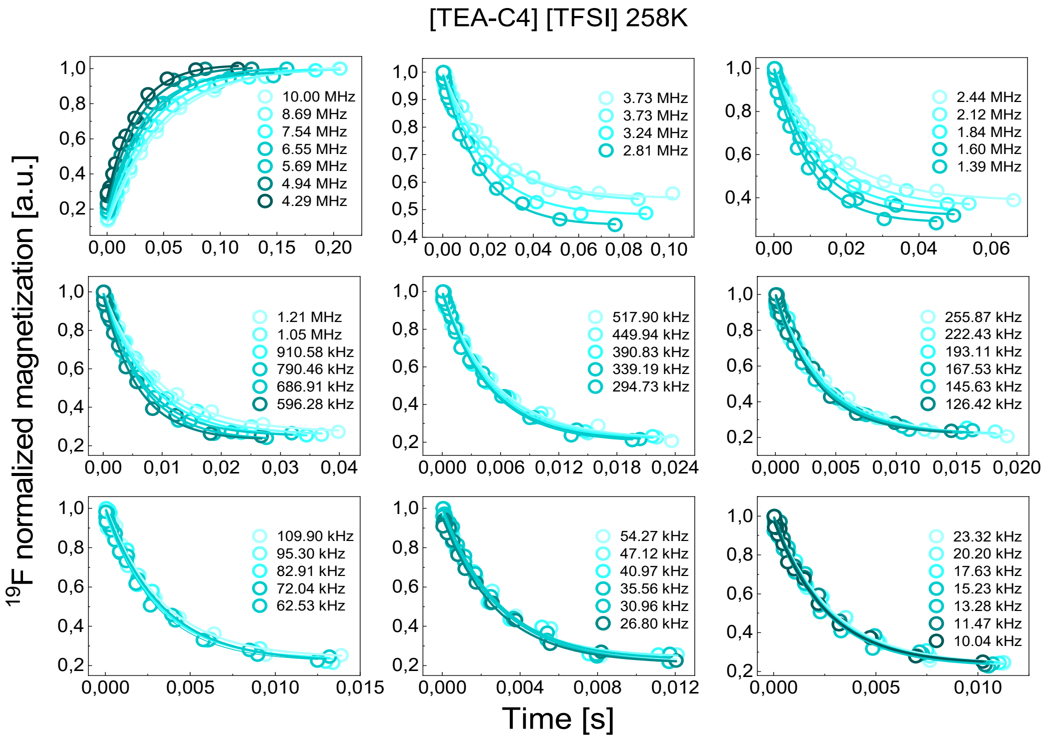

Figure S9. ^{19}F magnetization curves for [TEA-C4] [TFSI] at 258K.

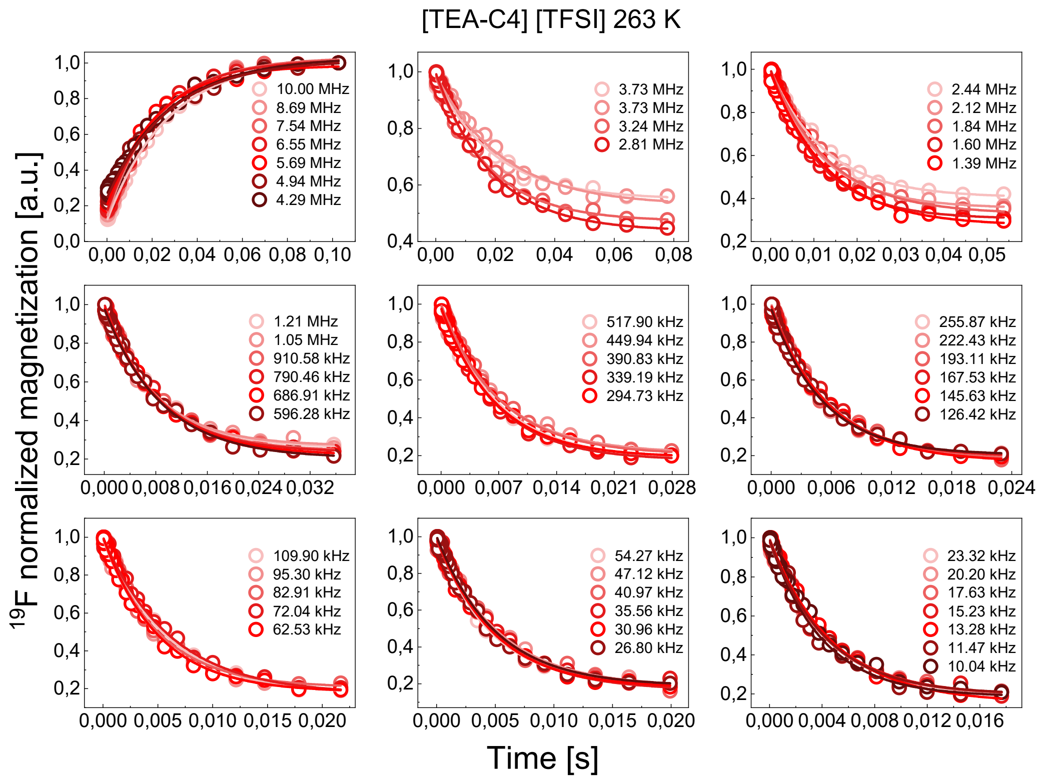

Figure S10. ^{19}F magnetization curves for [TEA-C4] [TFSI] at 263K.

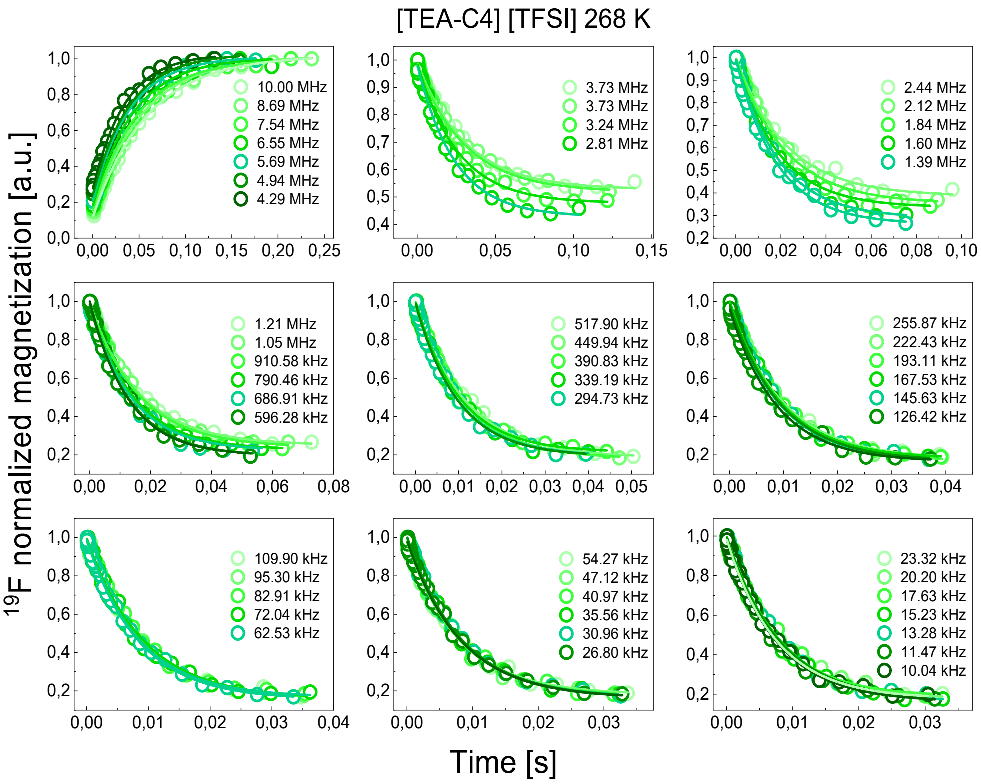

Figure S11. ^{19}F magnetization curves for [TEA-C4] [TFSI] at 268K.

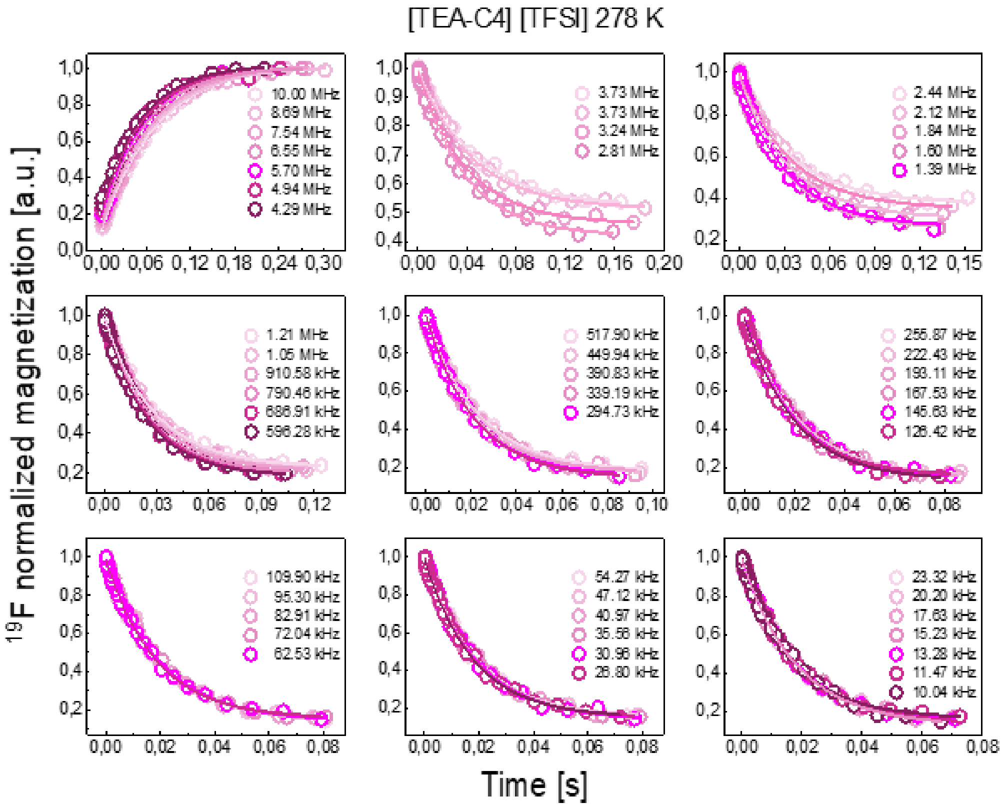

Figure S12. ^{19}F magnetization curves for [TEA-C4] [TFSI] at 278K.

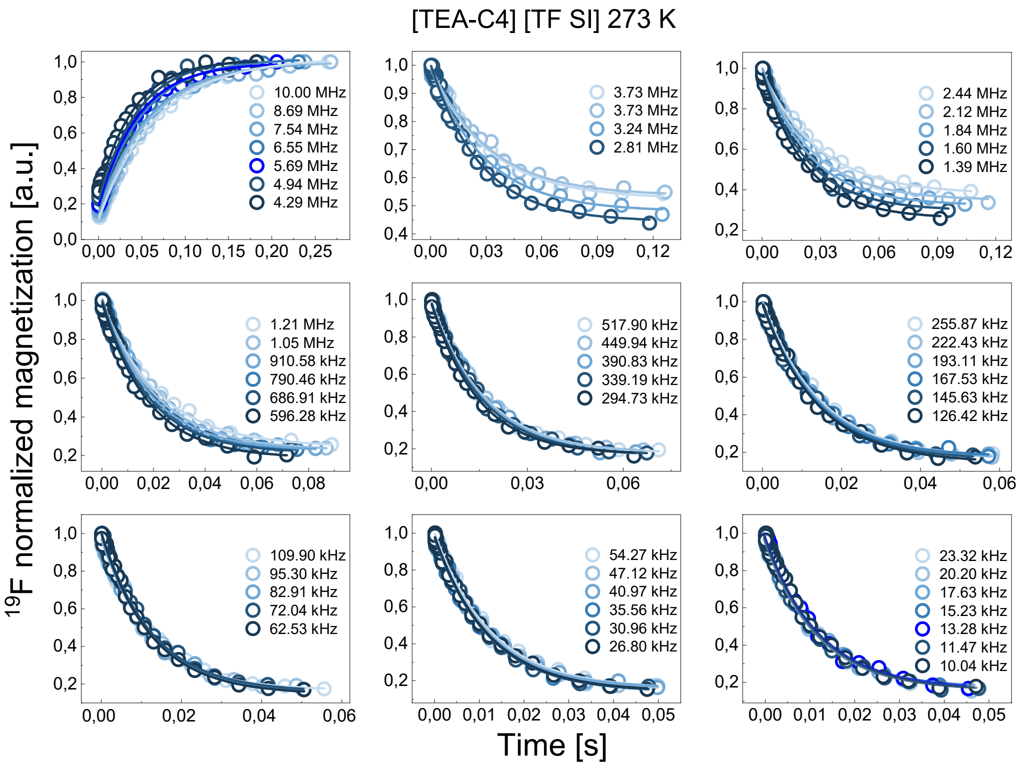

Figure S13. ^{19}F magnetization curves for [TEA-C4] [TFSI] at 273K.

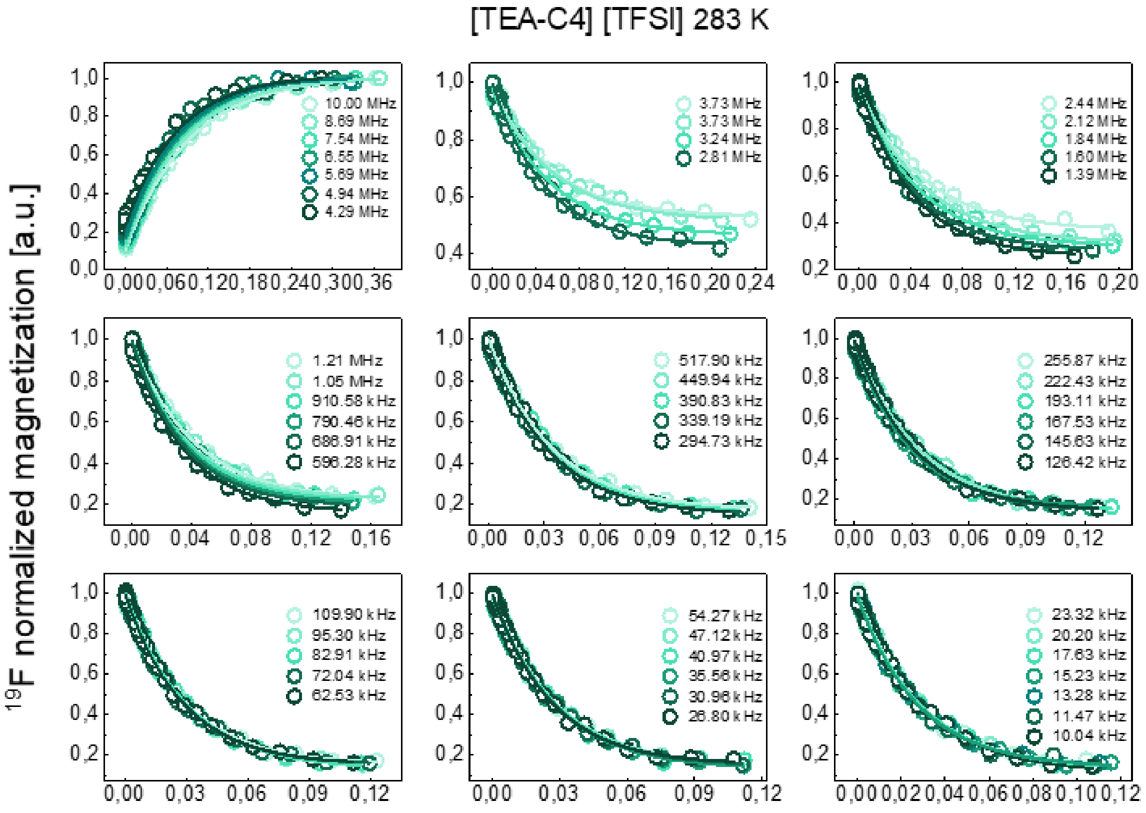

Figure S14. ^{19}F magnetization curves for [TEA-C4] [TFSI] at 283K.

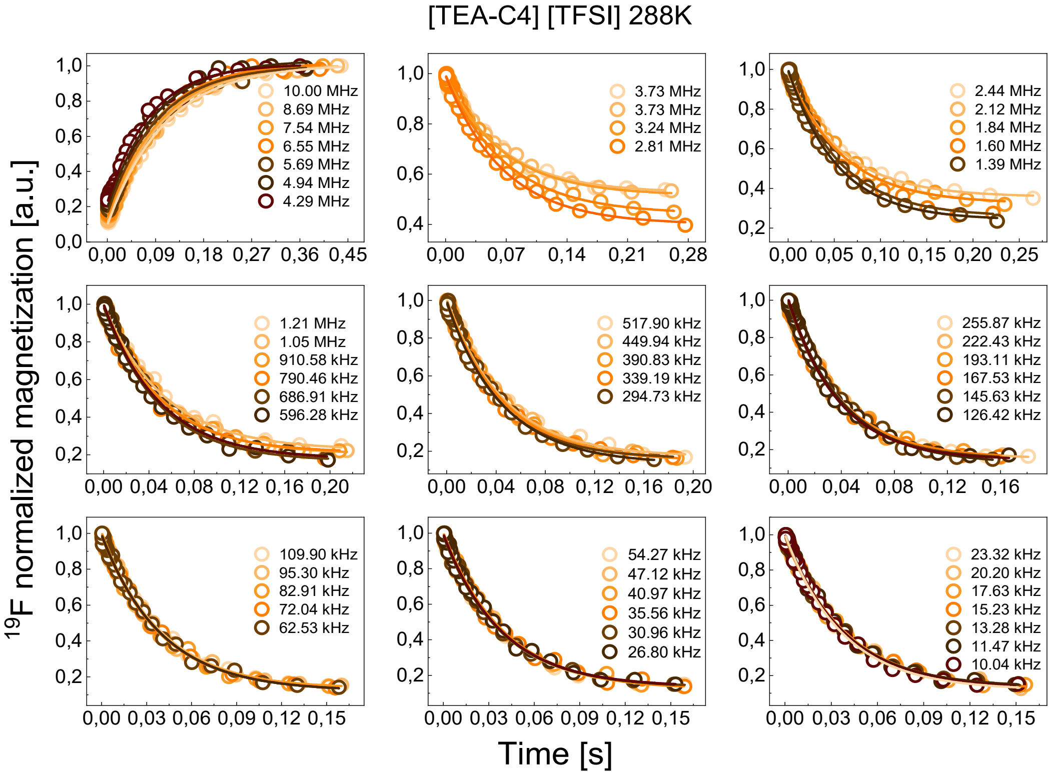

Figure S15. ^{19}F magnetization curves for [TEA-C4] [TFSI] at 288K.

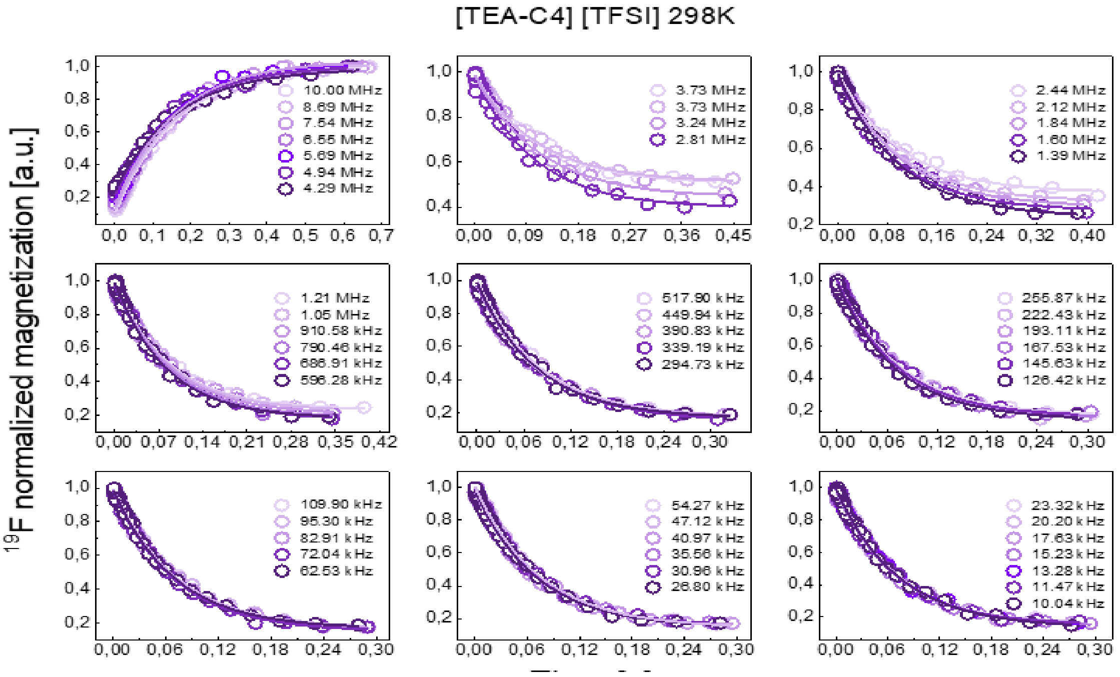

Figure S16. ^{19}F magnetization curves for [TEA-C4] [TFSI] at 298K.
